# Two Novel Cognitive Behavioral Therapy–Based Mobile Apps for Agoraphobia: Randomized Controlled Trial

**DOI:** 10.2196/jmir.7747

**Published:** 2017-11-24

**Authors:** Marina Christoforou, José Andrés Sáez Fonseca, Elias Tsakanikos

**Affiliations:** ^1^ Division of Psychiatry University College London London United Kingdom; ^2^ Department of Psychology University of Roehampton London United Kingdom

**Keywords:** agoraphobia, anxiety, eHealth, computerized interventions, mobile applications, randomized controlled trial, RCT

## Abstract

**Background:**

Despite the large body of literature demonstrating the effectiveness of cognitive behavioral treatments for agoraphobia, many patients remain untreated because of various barriers to treatment. Web-based and mobile-based interventions targeting agoraphobia may provide a solution to this problem, but there is a lack of research investigating the efficacy of such interventions.

**Objective:**

The objective of our study was to evaluate for the first time the effectiveness of a self-guided mobile-based intervention primarily targeting agoraphobic symptoms, with respect to a generic mobile app targeting anxiety.

**Methods:**

A Web-based randomized controlled trial (RCT) compared a novel mobile app designed to target agoraphobia (called Agoraphobia Free) with a mobile app designed to help with symptoms of anxiety in general (called Stress Free). Both interventions were based on established cognitive behavioral principles. We recruited participants (N=170) who self-identified as having agoraphobia and assessed them online at baseline, midpoint, and end point (posttreatment) over a period of 12 weeks. The primary outcome was symptom severity measured by the Panic and Agoraphobia Scale.

**Results:**

Both groups had statistically significant improvements in symptom severity over time (difference –5.97, 95% CI –8.49 to –3.44, *P*<.001 for Agoraphobia Free and –6.35, 95% CI –8.82 to –3.87, *P*<.001 for Stress Free), but there were no significant between-group differences on the primary outcome (difference 0.38, 95% CI –1.96 to 3.20, *P*=.64).

**Conclusions:**

This is, to our knowledge, the first RCT to provide evidence that people who identify as having agoraphobia may equally benefit from a diagnosis-specific and a transdiagnostic mobile-based intervention. We also discuss clinical and research implications for the development and dissemination of mobile mental health apps.

**Trial Registration:**

International Standard Randomized Controlled Trial Number (ISRCTN): 98453199; http://www.isrctn.com /ISRCTN98453199 (Archived by WebCite at http://www.webcitation.org/6uR5vsdZw)

## Introduction

People with agoraphobia have considerable impairment in their daily lives as a result of persisting avoidance of places and situations. Lifetime prevalence of agoraphobia with and without panic disorder in the adult population has been reported to range from 0.8% to 2.6% in American and European community samples [1,2]. Agoraphobia has been ranked as one of the most chronically persistent disorders [3], with higher rates of work-related inactivity and disability than with other chronic conditions [4,5].

The standard psychological treatment for agoraphobia symptoms is cognitive behavioral therapy (CBT), which is also recommended by the UK National Institute of Health and Care Excellence [6]. People with agoraphobia have catastrophic ideas regarding the likelihood of threat in a situation or environment, such as fainting and being ignored or ridiculed by others [7,8], and as a consequence engage in “safety behaviors” to prevent the expected catastrophe and reduce anxiety. Such safety behaviors preserve and might even enhance the maladaptive beliefs and thus maintain anxiety [9,10]. In CBT, cognitive restructuring is used to challenge catastrophic cognitions and unrealistic predictions, and to generate alternative, more realistic expectations. Behavioral experiments may be conducted to help disconfirm such beliefs and drop safety behaviors [8]. Moreover, imaginal or in vivo exposure to the feared situations is used to reduce situational avoidance and phobic anxiety. Breathing and relaxation exercises are sometimes employed to help the patient cope with overwhelming stress.

The effectiveness of CBT in the treatment of agoraphobia has been demonstrated in several studies. A systematic review and meta-analysis [11] examined the effectiveness of psychotherapy and pharmacotherapy in the treatment of panic disorder, with the majority of participants also having agoraphobia. They identified 23 randomized controlled trials (RCTs) and showed that, although combined psychotherapy and pharmacotherapy produced the best results in the short term, in the long term combined treatment was as effective as psychotherapy alone, and both treatment groups were superior to pharmacotherapy alone. Among the different types of psychotherapy, CBT had the strongest evidence. More recent studies have also provided support for CBT as an effective treatment in agoraphobia patients [12-15] in line with earlier studies [16].

Even though CBT has been established as the preferred and most effective treatment for agoraphobia and panic, many people remain untreated. From the population of people with anxiety disorders in developed countries, only 16.7% seek help from a mental health professional, and only 21.3% of those receive CBT [17]. Despite the fact that CBT has nowadays become more widely available, certain barriers to treatment may explain the low levels of self-seeking in people with agoraphobia. For example, fear of stigmatization, lack of psychoeducation, long waiting lists, transportation problems, and time constraints significantly limit access to CBT [18-20]. In addition, the very nature of agoraphobia, which may include fears of leaving the house and using public transport, may make it even more difficult for people to actively seek professional help. To overcome such barriers, effective interventions that are easily accessible and do not require therapist face-to-face contact should be available.

Internet-based or computerized interventions can be considered as acceptable alternatives to standard treatments, as they can be clinically effective and minimize treatment barriers for users. Such interventions can be self- or therapist guided, presenting materials of cognitive behavioral principles and methods in a series of lessons, which are typically accompanied by homework tasks and supplementary information. Systematic reviews show that computerized CBT (CCBT) is as beneficial as therapist-led CBT in the treatment of anxiety disorders such as panic disorder, and is more effective than treatment as usual or waiting list conditions [21,22]. CCBT can also reduce therapist time [21] and have good acceptability, as shown by adherence and satisfaction levels [22]. A more recent review of Internet CBT, including 8 trials of panic disorder with and without agoraphobia, concluded that Internet CBT was as efficacious as face-to-face CBT and more efficacious than waiting list, attention, information, and online discussion control groups [23].

One promising mode of delivering computerized interventions are mobile phones, because of their relatively low cost and widespread use [24]. Since users carry their mobile devices with them in almost any situation, mobile phones, and particularly smartphones, might facilitate engagement with exposure exercises in the users’ natural environments. Although mobile apps have been tested for several conditions, such as unipolar depression [25], borderline personality disorder, and substance abuse [26], to date there has been no study testing an app that primarily targets agoraphobic symptoms. Because of the isolative nature of agoraphobia and the extreme avoidance behaviors, an app that could be easily downloaded over the Internet onto patients’ phones or tablets, requiring no traveling to sites, might be especially appealing and convenient for this population.

This study was a Web-based RCT aiming to test the clinical effectiveness of a novel mobile app for agoraphobia in a community-based sample. The treatment app, Agoraphobia Free, developed by Health eLiving Partnership Ltd (HeLP) for the iOS and Android operating systems, provides an interactive game-based intervention using cognitive behavioral techniques that target agoraphobia and panic. The comparator was a stress-reduction app (Stress Free), which does not address agoraphobic symptoms or panic, but stress and anxiety in general. Both interventions were self-guided and were evaluated over a period of 12 weeks. Adults that self-identified with agoraphobia were randomly allocated to the 2 treatment arms and completed self-reported assessments at baseline, midpoint (6 weeks), and end point (12 weeks) of the trial. The primary objective was to examine whether an agoraphobia-specific intervention would be more effective than a generic, anxiety-related intervention. A secondary aim was also to assess the level of engagement with these interventions and the feasibility of conducting such a trial over the Web.

## Methods

### Design

The study was a Web-based, assessor-blinded, parallel-group RCT with an active control group. Participants were individually randomly allocated (ratio of 1:1) to either the treatment group or the control group at baseline and were given an equal amount of time to complete each intervention (12 weeks). Data were gathered automatically through online collection of anonymized data, without any researcher intervention. The primary outcome was the degree of symptom severity, as measured by the self-reported version of the Panic and Agoraphobia Scale [27] at end point.

### Participants

#### Inclusion Criteria

Participants needed to be adults (aged ≥18 years) and identify themselves as having agoraphobia. Participants also had to be willing and able to provide informed consent to participate in the trial.

We used no diagnostic check, as the aim was to recruit a community sample that would reflect the nature of the population that would use the apps in a real-world setting, where no screening or check would be required.

#### Exclusion Criteria

The exclusion criteria were as follows: (1) inability to give informed consent due to significant cognitive or intellectual impairment, (2) no adequate understanding of English as a first language, and (3) not having a mobile device than could run the app as designed.

### Recruitment and Setting

A website was set up for the trial on which advertisements and all relevant information and updates were posted. The website was hosted by HeLP Ltd (currently known as Thrive Therapeutic Software). Advertisements of the trial were also posted on social media (eg, Facebook, Twitter), support groups and forums, websites of relevant organizations such as Anxiety UK, blogs, and university websites. We also individually contacted members of anxiety support groups via Facebook or forum messages. We created a mailing list of people subscribing their interest in the trial, whom we encouraged to stay in touch until the trial commenced. Moreover, contacts of the Chief Investigator that were working in health-related settings were encouraged to inform any relevant clients or representatives about the study. There were 2 rounds of recruitment in order to achieve a larger number of participants.

We always contacted participants via email. Initially, those who expressed interest in participating followed a link to the online information sheet and consent form, which outlined the eligibility criteria and information about the trial. Through consenting to participate and answering a series of questions, participants confirmed that they met the criteria and understood the purpose of the study. Participants also provided their email addresses and names, though the latter was optional. Participant codes were then assigned to those who consented, and details about their mobile devices (smartphones or tablets) and demographics (age and sex) were obtained. Participants were instructed to use only their participant code to identify themselves.

Participants received invitations for the apps, which were available to use for free. Emails also contained links to weekly surveys on app use and links to questionnaires, a description of the specific survey questionnaire, links to the calendar and the main website of the trial, information about the upcoming survey, and useful contact details. A Frequently Asked Questions section was set up on the main website. Data were collected online and could only be accessed by the researchers.

### Interventions

#### Agoraphobia Free

The treatment app was Agoraphobia Free (version 0.8), developed by Thrive Therapeutic Software for the treatment of agoraphobia, and this was the first time that it was evaluated. The app was a game-based interactive intervention, with 3-dimensional characters and situations that simulate real-life environments. Specifically, the app presented a case example of a virtual character who had agoraphobia. The user was required to guide her, through the help of the virtual therapist, to complete the different therapeutic tasks. Those tasks were based on CBT principles, comprising psychoeducation, reflection, cognitive restructuring, interoceptive exposure, and systematic desensitization. The 3 overarching goals were to decrease the virtual character’s catastrophic cognitions, safety behaviors, and physiological arousal. In this way, users were able to progress through the character’s recovery and treatment, and build the formulation of her difficulties. At the same time, users were asked to apply the techniques they used in the case example to their own situation. Therefore, by using the character’s recovery journey as a template, users learned how to reduce their catastrophic thinking, their safety behaviors, and physiological arousal.

At the beginning of the intervention, we asked participants to set a hierarchy of goals they would like to achieve (eg, going out of the house, going to the supermarket). At the end of each session, they were required to complete each one of those goals in order, using the techniques and strategies they had learned in the intervention. The sessions were designed so that the tasks became increasingly more challenging as participants progressed through the intervention. The sessions needed to be completed in the order they were presented in for the next ones to become unlocked. There were 10 sessions in total, and the participants were asked to complete 1 or 2 sessions per week at their own pace, and reminders for those were sent weekly. Multimedia Appendix 1 shows an outline of each session.

#### Stress Free

The control app was Stress Free (version 1.3) developed by Thrive Therapeutic Software, which focused on teaching relaxation techniques and generic CBT skills though a virtual therapist. The app also included a few distraction techniques presented in the form of games that required attention to help individuals cope with acute anxiety. The intervention was presented initially as a linear training program using video and audio guides. The user first learned diaphragmatic breathing, then differential deep muscle relaxation, then self-hypnosis, and finally meditation. These relaxation techniques have been previously shown to be effective in reducing stress [28,29]. Participants rated their anxiety before and after relaxation sessions using a visual analog scale. After completing the training, they were familiarized with CBT concepts such as negative automatic thoughts and the process of challenging them. Finally, we gave participants a daily goal to complete, such as doing 3 sessions of diaphragmatic breathing or a 10-minute session of meditation. In between sessions, participants were prompted to record their anxiety on a CBT journal. The app only targeted stress and anxiety in general, so it was not specific to agoraphobia. In the original version, there was no maximum limit of sessions that users could go through until they mastered the techniques. However, for the purpose of the trial, we asked participants to complete 10 sessions in total, so as to match the number of sessions required from the treatment group.

Table 1 compares the 2 apps by showing which components and exercises were present in each. Although some components were common to both apps, in Agoraphobia Free they were specifically tailored to agoraphobia. In Stress Free the exercises addressed stress and anxiety in general without referring to agoraphobia. The 2 apps were matched for the number of sessions required and time to complete the interventions (minimum: 6 weeks, maximum: 12 weeks). No training, supervision, or guidance was offered before or during the trial, and only a basic description of each app was provided. Any questions participants had regarding the app or any technical issues they encountered were resolved through email. Weekly reminders and short surveys were sent to promote engagement and monitor progress, and the completion of 1 to 2 sessions per week was recommended for both groups. We also informed participants at the beginning of the trial that, when they completed the intervention, they would receive a link to download the app they did not receive for free, as a reward for taking part and an additional incentive to complete their assigned intervention. Participants were assured that they would not be asked any questions about the second app, as it would not be part of the research.

The 2 apps were available on Android and iOS. Multimedia Appendix 2 shows screenshots of the apps.

### Outcomes

The primary outcome was the severity of agoraphobic and panic symptoms, measured by the PAS [27]. The questionnaire was administered online in a self-report format at baseline, midpoint (6 weeks), and end point (12 weeks) of the trial. Reminders were emailed to those who did not reply to the questionnaires before the prespecified deadline. Participants rated the symptoms they experienced in the previous week on a 5-point scale. The questionnaire comprises 14 items, although only 13 of those are used to calculate severity scores. The items are grouped into 5 subscales: (1) Panic Attacks, assessing frequency, severity, and duration of panic attacks, (2) Agoraphobic Avoidance, assessing frequency of avoidance, and number and relevance of avoided situations, (3) Anticipatory Anxiety, assessing frequency and severity of anxiety, (4) Disability, assessing impairment in family life, social relationships, and employment, and (5) Worries about Health, assessing worries about damage to health and assumption of organic disease. The scale was originally validated in a sample of 235 panic patients and has shown good internal consistency (Cronbach alpha=.88), test-retest reliability, and good internal and external validity [27,30]. Cronbach alphas for this study’s sample indicated good internal consistency, with alpha=.84 for the overall scale. Coefficients for individual subscales ranged from .41 to .88. The scale has been shown to be sensitive to change due to treatment in 2 clinical trials [31,32].

The secondary outcomes were completion of the intervention and engagement with the apps. Completion of the interventions was assessed in the short online surveys that were sent weekly, by asking participants if they had used the app, how much time they used it for over the past week, and how many sessions they had completed. If participants claimed that they had not used the app, they were asked to give reasons.

### Sample Size

We estimated the sample size on the basis of using the self-rated version of the PAS (SD 10.3) as the primary outcome measure. At least 68 participants in each arm were needed to detect a 5-point between-group difference, with a 2-sided significance level set at 5% and power at 80%. Given the high dropout rates in Web-based trials [33], the aim was to recruit at least 150 participants in total.

**Table 1:** The different features present (indicated by “X”) in each app.

App features	Stress Free app	Agoraphobia Free app
Relaxation technique training	X	X
Automated activity goals	X	X
CCBT^a^ basic tutorial	X	X
CCBT journal with prompts	X	X
Maintenance sessions	X	X
Self-soothing strategies	X	X
Distraction techniques	X	X
Structured CCBT program		X
Goal setting by user		X
Construction of exposure hierarchy		X
Development of a formulation		X
Relapse prevention session		X

^a^CCBT: computerized cognitive behavioral therapy.

### Randomization

We used a random computer-generated sequence to randomly allocate participants to the 2 intervention groups. The random allocation sequence was retrieved from a website that generates truly random numbers [34], by a person outside the research team. We applied block randomization to ensure equal numbers of participants in each group (ratio of 1:1). Participants were automatically allocated to intervention groups by a formula on Excel version 14.6.6 (Microsoft Corporation) using the random number sequence, which was coordinated by another contact who was not a member of the research team. The random sequence and the allocation of participants to groups were concealed from research staff throughout the trial. The same person sent emails to participants containing the link to the assigned app after they had returned the baseline questionnaire.

### Blinding

The trial was assessor blinded, as researchers were blinded to treatment allocation throughout the trial and during the statistical analysis. This was achieved by having a person outside the research team to manage treatment allocation and personal communications with the participants. Any questions or comments made in the surveys were forwarded from this contact to research staff, excluding any participant details or codes. This was to ensure that the researchers remained blinded to treatment allocation, as some comments contained information about the specific app the participants were using. Researchers did not have any access to data regarding treatment allocation, as those were stored on a secure database, separate and protected from other research files. During data collection and analysis, only numerical codes were used to indicate group allocation. Researchers did not know which groups those referred to until the end of the analysis.

Blinding the participants was not possible, as the apps were clearly labelled as “Stress Free” and “Agoraphobia Free,” and masking those would require significant changes in the software, which were not feasible. Moreover, even if the apps were not differentially labelled, it is very likely that participants would have become aware of which treatment group they were assigned to because of the intervention content and the extent to which it addressed agoraphobia.

### Ethics

The research was approved by the Roehampton University Ethics Committee (reference number: PSYC 14/ 117). The trial was registered and reported in accordance with the Consolidated Standards of Reporting Trials (CONSORT)-EHEALTH checklist (Multimedia Appendix 3) [35]. Data were kept anonymized and protected according to the UK Data Protection Act [36]. We could obtain information about adverse events or effects of the interventions from feedback participants provided in the weekly surveys.

### Analysis

We analyzed the data on Stata version 14 (StataCorp LLC). We checked baseline data for normality and obtained descriptive statistics to capture the demographic and clinical characteristics of the sample. The analysis performed was intention-to-treat, as requested in the CONSORT [37]. All participants who completed the baseline assessment were included in the analysis as they were randomly allocated.

We used a linear mixed model to analyze the data, with a random effect of participant, and fixed effects of time (baseline, midpoint, end point), group (Agoraphobia Free and Stress Free), and the interaction between time and group. The estimated baseline PAS score was constrained to be identical in the 2 groups, thus adjusting for baseline and allowing the relationship between baseline and follow-up scores to differ at each time point. Another advantage of this statistical method is that the data from all participants contribute to the analysis, even if there is a substantial amount of missing data at follow-up [38]. We used an unstructured residual covariance matrix to allow for correlations within participants between the different time points. Statistical significance was taken at the 5% level (*P*<.05).

We conducted a planned secondary completers’ analysis using the same data analytic strategy as the intention-to-treat analysis. This analysis included only those participants who were identified as intervention completers.

## Results

### Participant Flow

**Figure 1:**
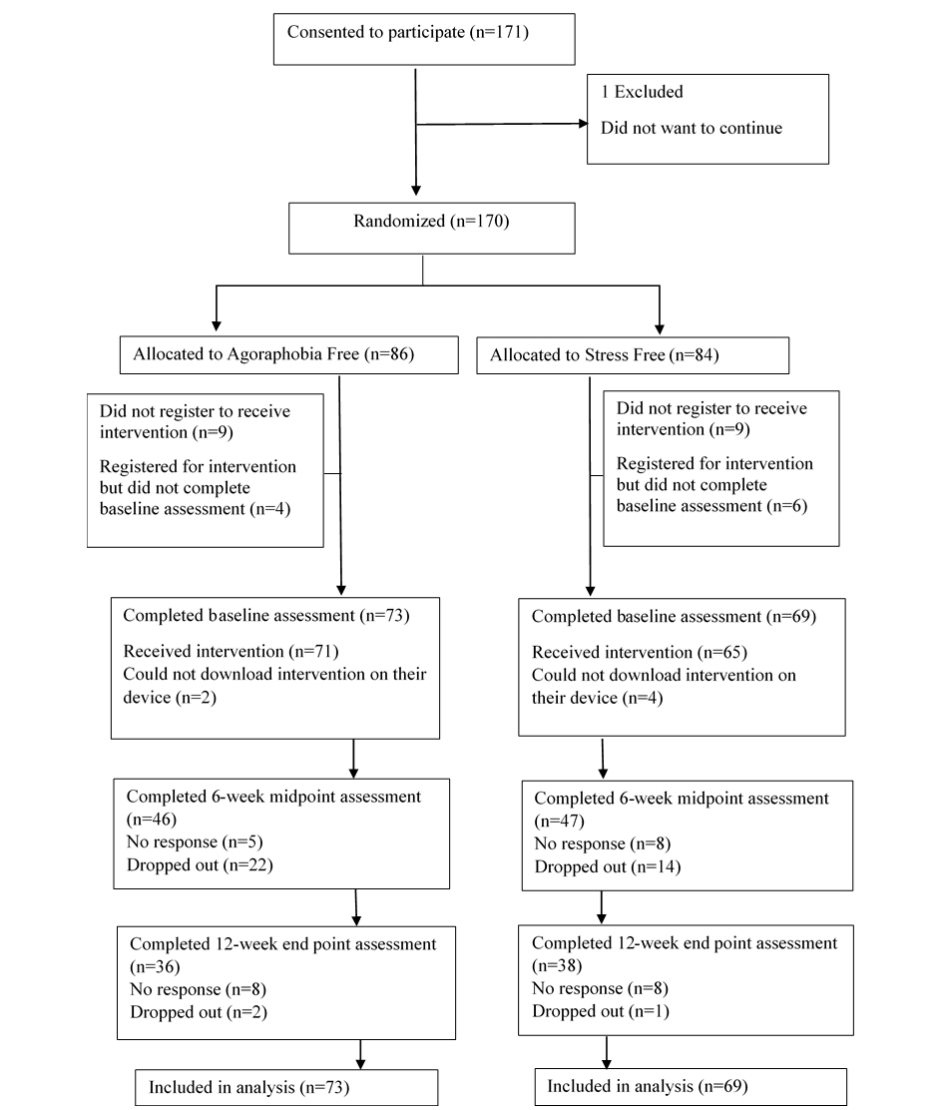
Flow of participants through the different stages of the trial.

The first phase of recruitment started from September 2014 and ended in late February 2015, and the second phase started in March 2015 and concluded in April, 2015. In the first round, 153 participants consented to participate, and we recruited 17 additional participants in the second round. The procedure following recruitment was the same in both samples. Data collection ended in June 2015. Figure 1 shows how the total sample of 170 participants progressed through the trial.

After 171 individuals consented to participate, 1 wanted to withdraw from the trial. The rest were randomly allocated into the 2 arms, but were not told which app they were assigned to at that point. Although the aim was to assign equal numbers of participants to each group, at each stage of randomization, the treatment group happened to have 1 more person than the other group, because the number of participants recruited each time was odd, and researchers were blinded to the allocation sequence.

We asked participants to fill in a form with their demographic and device details to register for the intervention so that we could set up the corresponding app invitations. A total of 18 participants did not return the form and did not reply to emails, even after they had been sent several reminders; thus, we treated them as dropouts and excluded them from the trial. The baseline PAS questionnaire was then sent and had to be completed in order for participants to proceed and receive the intervention. A total of 10 individuals did not return the questionnaire and did not reply to any emails. Up to this point, participants were still unaware of the group they were assigned to; thus, their exclusion from the trial was very unlikely to introduce any bias. The remaining 142 who completed the PAS were sent their allocated intervention, but 6 participants could not download the app on their device, despite efforts to resolve the technical issues.

After baseline, 39 participants did not want to continue with the trial and dropped out, while 29 participants did not reply to emails and did not complete the questionnaires. We included all participants who provided baseline data in the analysis.

### Baseline Characteristics

Of the 142 participants who completed the baseline assessment, 2 did not give details of their age and sex (1 from each treatment arm). Of the remaining 140, 118 (84.3%) were female and had a mean age of 39.7 years (SD 11.3). Overall, the mean participant PAS total score was 30.3 (SD 8.7), and the scores were normally distributed (Shapiro-Wilk test: *W*=.985, *P*=.14).

The mean PAS total score fells into the severe range and was much higher than that of the original sample used in the validation of the scale [27], which reported a mean score of 23.5, SD 10.3. Our sample also had a higher proportion of women than the original sample (of whom only 57% were women) and a slightly higher age (mean 36.09 years in the original sample). A significant proportion of our sample (114/142, 80.3%) had severe or extremely severe Agoraphobic Avoidance, while 85.2% (121/142) avoided more than 8 situations. None of the participants scored 0 on the Agoraphobic Avoidance subscale, which indicates that all of them experienced agoraphobic symptoms to some degree. The lowest score on that scale was 1.67, indicating mild Agoraphobic Avoidance. In contrast, 29 participants (20.4%) obtained a score of 0 on the Panic Attacks subscale, which suggests that some participants experienced agoraphobia in the absence of panic attacks. Overall, agoraphobic symptoms were more prominent than panic symptoms in this sample.

Table 2 shows the demographic and clinical baseline characteristics of the participants by group. The 2 groups did not differ statistically on any of those characteristics at baseline (all *P*>.05).

### Missing Data

A total of 68 (47.9%) participants had missing outcomes. Overall, the differences between participants with missing data and those without were not statistically significant on any of the baseline variables examined. There were no significant differences in age (*t*_138_=0.85, *P*=.40) sex (χ^2^_1_=0.6, *P*=.45), or clinical symptom severity (*t*_140_=1.32, *P*=.19). There were 37 participants (54%) with missing data in the Agoraphobia Free arm and 31 (46%) in the Stress Free arm. The relationship between missing data and treatment arm was not significant (χ^2^_1_=0.5, *P*=.49). Therefore, participant attrition did not seem to be biased with regard to group or any other baseline factor.

### Main Analysis

We produced a linear mixed model assessing the relative effects of each intervention on PAS scores at the 2 follow-up time points. Table 3 presents the estimated differences in PAS scores for the Agoraphobia Free group compared with the Stress Free group adjusted for baseline score at the 2 time points.

**Figure 2:**
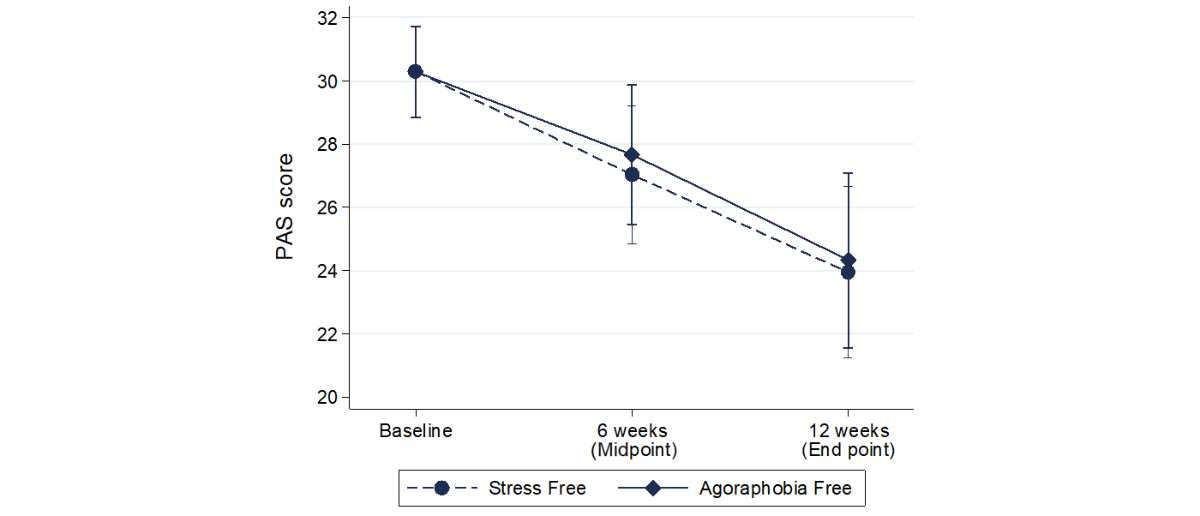
Clinical symptom severity as indicated by the total score on the Panic and Agoraphobia Scale (PAS) in each group at trial baseline, midpoint, and end point.

At end point, symptom severity scores decreased in both groups, but there was no evidence that the changes were significantly greater among participants of the Agoraphobia Free group than among those in the Stress Free group. Similarly, at midpoint there were no significant differences in symptom severity changes between the 2 groups. Therefore, there were no significant differences between the 2 groups. Figure 2 presents the differences on the primary outcome for each group over time.

We carried out the same linear mixed model analysis (n=142) using each PAS subscale as the dependent variable to examine whether there was a difference between the 2 groups in terms of symptom dimensions. We found no significant interactions between group and time for any of those outcomes (all *P*>.05).

We conducted within-group contrasts to examine the degree of change in symptom severity over time. For both Agoraphobia Free (n=73) and Stress Free (n=69), there were statistically significant improvements in symptom severity from baseline to midpoint and end point. Table 4 presents the results.

**Table 2:** Baseline demographic and clinical characteristics of each treatment arm.

Characteristics		Agoraphobia Free app (n=73)	Range of PAS^a^ scores	Stress Free app (n=69)	Range of PAS scores
Age (years), mean (SD)		39.21 (10.45)	N/A^b^	40.23 (12.21)	N/A
Sex (female), n (%)		64 (88.9)	N/A	54 (79.4)	N/A
**PAS scores, mean (SD)**					
	Total	30.77 (8.72)	9-50	29.80 (8.72)	6-47
	Panic Attacks	1.58 (1.05)	0-4	1.52 (0.88)	0-3.33
	Agoraphobic Avoidance	3.30 (0.57)	1.67-4	3.24 (0.52)	2-4
	Anticipatory Anxiety	2.70 (0.94)	0-4	2.59 (1.01)	0-4
	Disability	2.39 (1.16)	0-4	2.47 (1.09)	0-4
	Worries about Health	1.78 (1.17)	0-4	1.46 (1.11)	0-4

^a^PAS: Panic and Agoraphobia Scale.

^b^N/A: not applicable.

**Table 3:** Intention-to-treat analysis at end point (12 weeks) and midpoint (6 weeks), n=142 *.*

Time point of PAS^a^ score	Agoraphobia Free mean (SD)	Stress Free mean (SD)	Effect estimate		
			Difference	95% CI	*P* value
End point (primary outcome)	24.33 (16.81)	23.95 (16.51)	0.38	–1.96 to 3.20	.64
Midpoint	27.66 (13.37)	27.03 (13.27)	0.62	–3.13 to 3.89	.83

^a^PAS: Panic and Agoraphobia Scale.

**Table 4:** Within-group contrasts capturing the differences in Panic and Agoraphobia Scale total score between time points within each treatment arm (n=142).

Treatment arms	Baseline-midpoint contrast			Baseline-end point contrast		
	Difference	95% CI	*P* value	Difference	95% CI	*P* value
AF^a^	–2.64	–4.48 to –0.79	.005	–5.97	–8.49 to –3.44	<.001
SF^b^	–3.25	–5.09 to –1.43	<.001	–6.35	–8.82 to –3.87	<.001

^a^AF: Agoraphobia Free app.

^b^SF: Stress Free app.

### Completers’ Analysis

Data on completion were available from participants who consistently replied to the surveys and questionnaires (n=74). There were 56 participants who completed 80% or more of the assigned intervention; 25 of those received Agoraphobia Free and 31 received Stress Free. Of those who were deemed noncompleters, 11 were in the Agoraphobia Free arm and 7 in the Stress Free arm. There was no relationship between treatment arm and completion of intervention (χ^2^_1_=1.5, *P*=.22).

We examined differences in completers’ symptom severity between the 2 intervention groups. We produced a linear mixed model, with random effect of participant, and fixed effects of time (baseline, midpoint, end point), group (Agoraphobia Free and Stress Free), and the interaction between time and group. In line with the intention-to-treat analysis, there were no significant differences between the 2 groups at end point or midpoint, as Table 5 shows. The within-group changes at each follow-up time point compared with baseline were significant in both groups (all *P*<.001). Therefore, there were significant reductions in symptom severity in both groups over time, but those reductions were equivalent across the 2 groups.

### Clinical Significance

Wichmann et al [39] recently showed that an overall postintervention (ie, face-to-face CBT) decrease in the total PAS score of about 4 to 5 points represents a clear clinical change in terms of quality of life and functioning. This is slightly smaller than the change we observed in our study (6 points) for end point PAS scores compared with baseline. The decrease in symptom severity was even more marked in the completers’ sample (7 points in the Stress Free group and 10 points in the Agoraphobia Free group). Therefore, it seems that both apps were overall successful in achieving clinically significant change.

Of the 74 participants who provided data at end point, 46 (62%) had a reduction of 5 or more points on the PAS at end point. A total of 25 (66%) participants in the Stress Free group and 21 (58%) participants in the Agoraphobia Free group improved at least 5 points on the PAS at end point.

### Safety and Use of Apps

A total of 7 participants (Agoraphobia Free: n=3, Stress Free: n=4) commented that certain app components were mildly stressful (eg, the distraction games, background music). There were no reported adverse events experienced as a result of either intervention. Also, 7 participants commented that the Agoraphobia Free app was confusing to follow at certain points. Another participant explained that they could not use the treatment app as much as they wanted to because they had depression and did not feel motivated.

**Table 5:** Completers’ analysis at end point (12 weeks) and midpoint (6 weeks), n=56.

Time point of PAS^a^ score	Agoraphobia Free mean (SD)	Stress Free mean (SD)	Effect estimate		
			Difference	95% CI	*P* value
End point	21.58 (13.10)	23.73 (12.12)	–2.15	–6.21 to 1.91	.30
Midpoint	26.09 (11.15)	26.93 (10.40)	–0.84	–4.05 to 2.37	.61

^a^PAS: Panic and Agoraphobia Scale. The mean baseline PAS score for both groups was 31.23.

## Discussion

### Principal Findings

The results from this RCT showed that participants who received Agoraphobia Free did not improve more than those who received the Stress Free app. Both groups showed reductions in symptom severity over time that were statistically significant, but those reductions seemed to be equivalent across the 2 groups. Both treatment apps were safe to use and yielded similar completion rates. Moreover, completers of either intervention showed marked improvements in symptom severity, with a 10-point drop in the PAS at end point compared with baseline in the Agoraphobia Free group. However, findings from this analysis should be considered with caution, as we performed the analysis on a specific subgroup of participants, and there is the possibility that factors other than the intervention influenced the outcome (eg, participant expectations). In addition, the power of this analysis was very low (below 50%) due to the small sample size.

Throughout the trial, participant attrition was particularly high, as almost half of the participants recruited dropped out of the trial or stopped responding to emails. Dropout rates were similar to those reported in other Web-based trials of self-guided interventions [40,41]. Many participants dropped out after providing consent, which we had not expected. We had to exclude those participants from the analysis, as no baseline data were available. It is unlikely that this exclusion of participants could have led to bias, as participants at that point were unaware of the groups they were randomly allocated to, and thus attrition was random between the 2 groups. Similarly, participants who dropped out or stopped responding after receiving the intervention did not seem to differ from those who engaged in the trial, and attrition was equivalent across the 2 groups. The analytic strategy we chose is robust and uses all available data from each participant, producing less-biased results than other methods of analysis and data imputation [38]. Overall, the fact that participant attrition appeared random, in addition to the fact that we used data from all participants in the analysis, offer support and confidence in the validity of the study findings.

### Limitations

The trial had a few limitations. There was no follow-up after the completion of the intervention. Therefore, it was not possible to investigate whether the improvements in symptom severity were maintained over time or whether there were any differences between the 2 groups after a few months of using the apps. The information collected regarding the characteristics of the sample was also limited, as there were no data on comorbid disorders, other psychological or physiological treatments, or other demographic characteristics such as ethnicity and computer literacy. This information might have provided better insights into the sample and could have been related to intervention efficacy. There was also limited information on app completion, as the information available relied on participant report and we were not permitted to extract app use data for participants individually. Moreover, participants could not be blinded, which is a common limitation in eHealth trials.

Another limitation of the study was the absence of a waitlist control group. The comparison of 2 active groups that shared very similar features tested the effects of agoraphobia-specific therapeutic elements over and above those of a generic anxiety-related treatment. There was no control, however, for the effect of time, and it is therefore not possible to conclude whether the improvements observed were not because of natural recovery processes or factors other than the intervention. Although a waitlist control group would be a necessary addition in a future trial in order to clearly establish treatment efficacy, the primary focus of this study was to demonstrate whether a disorder-specific mobile-based intervention is warranted in the treatment of agoraphobic symptoms, compared with a more generic approach addressing anxiety.

Despite its limitations, the study also presented certain strengths. First, it is, to our knowledge, the first RCT to directly compare an agoraphobia-specific app with a generic anxiety-related app. Second, the sample recruited online was characterized by severe clinical symptoms and especially high levels of agoraphobia, which shows that recruitment based on self-identification is feasible and reliable. Such a sample may have also been hard to reach in a traditional multicenter trial with assessors conducting screening interviews, which would have been time consuming and would require a lot of resources. The trial was minimally intrusive for the participants, as it requested a limited amount of information, and the tools used were short and easy to complete. This helped achieve fair recruitment and response rates without any explicit individual guidance. Third, an important strength of the study was its external validity. The apps were offered as they would be offered in real-world settings, without requiring any screening of users or close supervision. There were very few restrictions on who could participate, therefore making our findings more easily generalizable to an Internet population who would simply download the app if they thought it would be relevant to them. Fourth, the results of the study provide an insight into what we would realistically expect from an unguided mobile intervention, such as a substantial dropout rate, small symptom change, and highest efficacy for those who consistently engage with and complete the intervention.

### Comparison With Prior Work

The findings of this study relate to previous studies on the treatment of anxiety disorders. The findings add to the evidence base of computerized cognitive behavioral interventions examined in anxiety disorders [21-23]. Our findings are also consistent with previous studies showing that completers of Web-based interventions for panic (with or without agoraphobia) benefit the most [40,42]. Importantly, the lack of a significant difference between the 2 groups indicates that a generic anxiety-related app and a diagnosis-specific app are equally effective in treating agoraphobic symptoms. This finding is in line with evidence supporting the efficacy of transdiagnostic (ie, unified, nonspecific) cognitive behavioral treatments in anxiety disorders. Transdiagnostic treatments are based on the premise that “commonalities across disorders outweigh the differences” [43]. A recent systematic review and meta-analysis showed that transdiagnostic treatments can be as efficacious as diagnosis-specific treatments in reducing anxiety symptoms and more effective than waitlist or attention control conditions [44]. Transdiagnostic CBT programs tested in different anxiety disorders (including panic disorder) can also be successfully administered via the Internet [45], producing effects equivalent to those of disorder-specific Internet-based interventions [46,47]. Moreover, transdiagnostic CBT does not differ from diagnosis-specific CBT in terms of treatment credibility [48]. Our study adds to this body of evidence suggesting that, for people with agoraphobia, an agoraphobia-specific app does not produce any additional benefits in relation to a transdiagnostic anxiety-targeting app.

### Clinical Implications

The findings of this study have implications for clinical practice. This trial shows that mobile apps can be successfully administered to a particular population that is hard to reach otherwise, without requiring guidance by a clinician. This could potentially save time for clinicians, while it could also be more convenient for patients and help them overcome many barriers to treatment, such as traveling to sites or long waiting lists. Furthermore, a self-guided app could be easily introduced into a stepped care model as a minimal intervention, as it is less intense than a clinician-guided intervention or individual therapy [49]. Since there was no evidence of superiority of one type of intervention over the other in this study, a choice between a generic and a targeted approach could be ultimately based on clinical judgment or patient preference. However, as suggested by Norton and Barrera [48], a generic anxiety-targeting app might be more resource efficient and easier to implement and disseminate than a diagnosis-specific app.

### Conclusions

Overall, this RCT suggests that transdiagnostic anxiety-targeting mobile apps can be as effective as disorder-specific apps for people with agoraphobic symptoms. Results show that individuals who identify as having agoraphobia do not benefit more from an agoraphobia-specific than from a transdiagnostic app. Future research conducted by independent research teams should replicate the results of this study, further investigating the possibility that mobile-based transdiagnostic interventions for anxiety can be as effective as current gold standard disorder-specific interventions. A trial with a larger sample size and a waitlist control group is warranted to establish intervention efficacy and cost effectiveness in this population. Additionally, the collection of more-extensive demographic and clinical information can help examine under which circumstances a diagnosis-specific or a generic approach is more appropriate. For example, future research could investigate whether patients with more severe symptoms or with comorbid disorders (eg, depression) benefit more from one type of intervention than the other. While there is still much to learn about treatment approaches in anxiety disorders, many studies, including this one, show that e-mental health interventions can overcome barriers and be effective in reducing clinical symptoms.

## Appendix

Multimedia Appendix 1 Agoraphobia Free session outline.

Multimedia Appendix 2 Screenshots of Agoraphobia Free and Stress Free.

Multimedia Appendix 3 CONSORT-EHEALTH checklist (V 1.6.1).

## Abbreviations

CBT: cognitive behavioral therapy
CCBT: computerized cognitive behavioral therapy
CONSORT: Consolidated Standards of Reporting Trials
HeLP: Health eLiving Partnership Ltd
PAS: Panic and Agoraphobia Scale
RCT: randomized controlled trial
