# Supplementary material for: Two Novel Cognitive Behavioral Therapy–Based Mobile Apps for Agoraphobia: Randomized Controlled Trial
Source: J Med Internet Res. 2017 Nov 24;19(11):e398. doi: 10.2196/jmir.7747 (PMC5722980; doi:10.2196/jmir.7747)
Supplement: Multimedia Appendix 1 [file jmir_v19i11e398_app1.pdf]

Appendix 1 - Agoraphobia Free session outline

Session 1

-Diaphragmatic breathing training
-Explanation of exposure therapy
-Explanation of using diaphragmatic breathing when encountering anxiety as part of exposure
-Creation of hierarchy
-Once the hierarchy is established the user is able to track progress against it. The user is prompted to tackle each stage of the hierarchy at his/her own pace throughout all the following sessions.

Session 2

-Explanation of common cognitive distortions
-Introduction to journaling
-Explanation of automatic negative thoughts with interactive example
-Introduction of the concept of formulation

Session 3

-Explanation of the concept of distraction or shifting attention from anxiety-inducing thoughts
-Practice identifying automatic negative thoughts and cognitive distortions
-Practice identifying physical responses to negative thoughts
-Practice diaphragmatic breathing
-Practice a distraction technique
-Practice completing a journal entry

Session 4

-Training in progressive deep muscle relaxation
-Practice identifying negative thoughts and cognitive distortions, physical sensations and resulting actions
-Practice distraction techniques
-Practice completing a journal entry
-Practice diaphragmatic breathing

Session 5

-Training using music as a distraction

-Practice identifying negative thoughts and cognitive distortions, sensations and actions
-Practice completing a journal entry
-Practice with sample formulations using a case study

Session 6

-Training to use self-suggestion/visualisation as an acute anxiety management technique
-Explanation of challenging cognitive distortions and automatic negative thoughts with practical exercise
-Practice completing a journal entry
-Training on how to start completing a self-formulation based on previous practice

Session 7

-Training on meditation
-Practice formulation with a case vignette
-Practice completing a journal entry including challenging negative thoughts

Session 8

-Practice formulation with a case vignette
-Progress on self-formulation if not completed
-Practice completing a journal entry
-Practice one relaxation technique

Session 9

-Practice one relaxation technique
-Progress on self-formulation if not completed
-Complete a journal entry

Session 10

-Full review of all content
-Full recap on progress to date
-Goal is to complete the hierarchy if not done already
-Relapse prevention plan

The app can then be used as a CBT journal and all relaxation and distraction techniques are available for the user to choose freely.
