# Supplementary material for: Two Novel Cognitive Behavioral Therapy–Based Mobile Apps for Agoraphobia: Randomized Controlled Trial
Source: J Med Internet Res. 2017 Nov 24;19(11):e398. doi: 10.2196/jmir.7747 (PMC5722980; doi:10.2196/jmir.7747)

Appendix 2 – Screenshots of Agoraphobia Free and Stress Free

Agoraphobia Free (A-C) and Stress Free (D-F)

A.

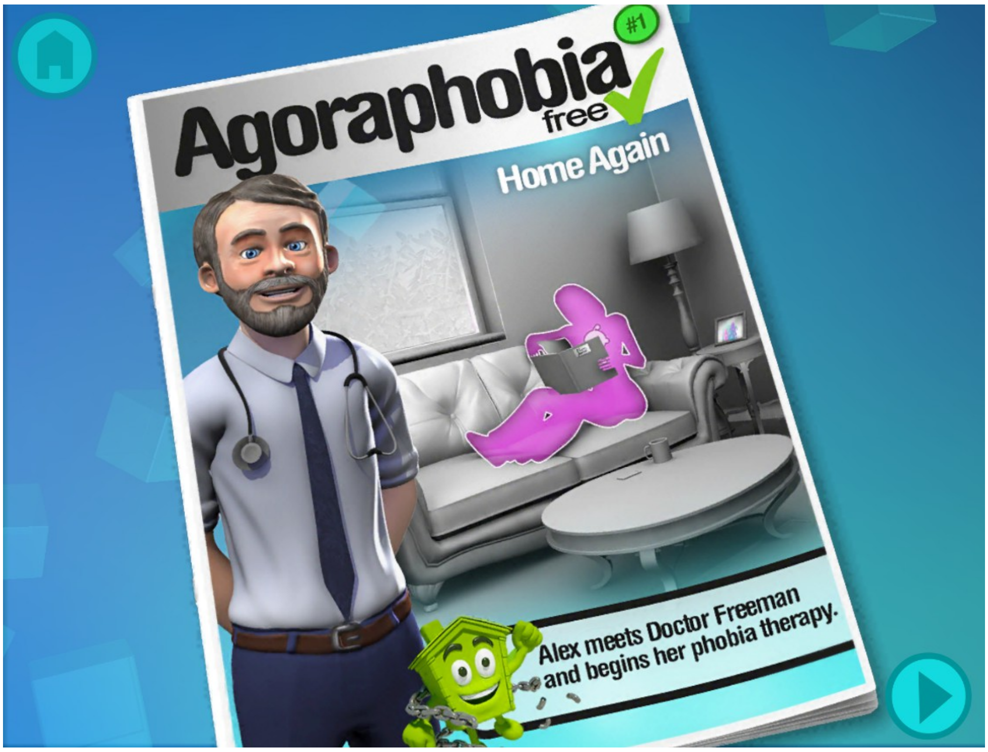

B.

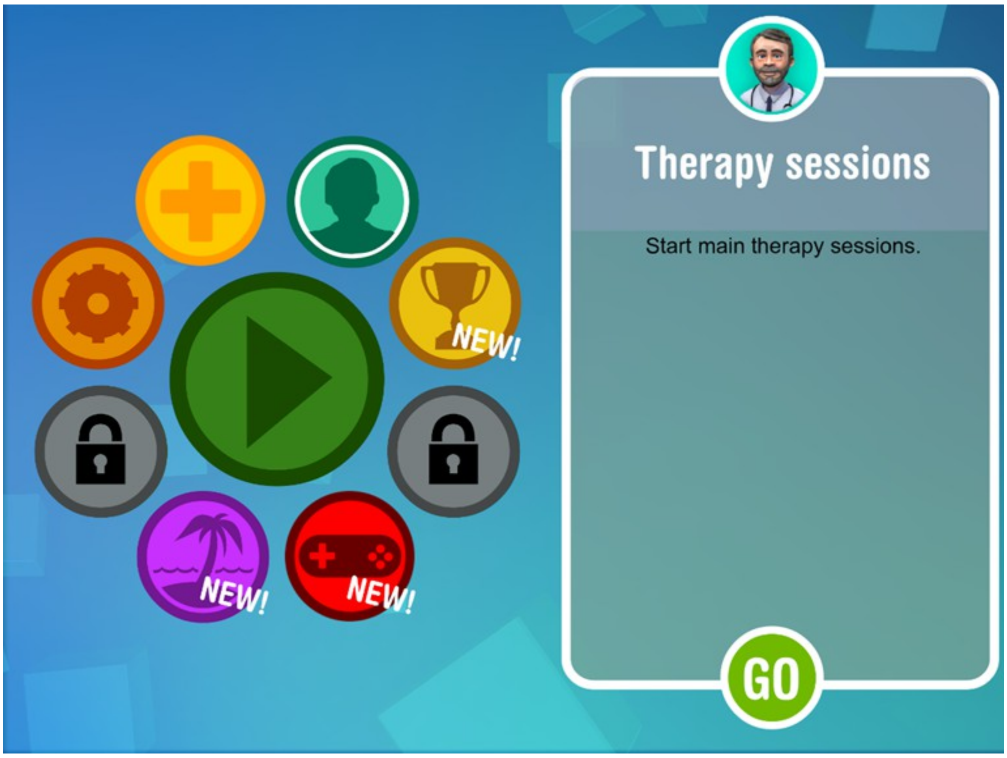

C.

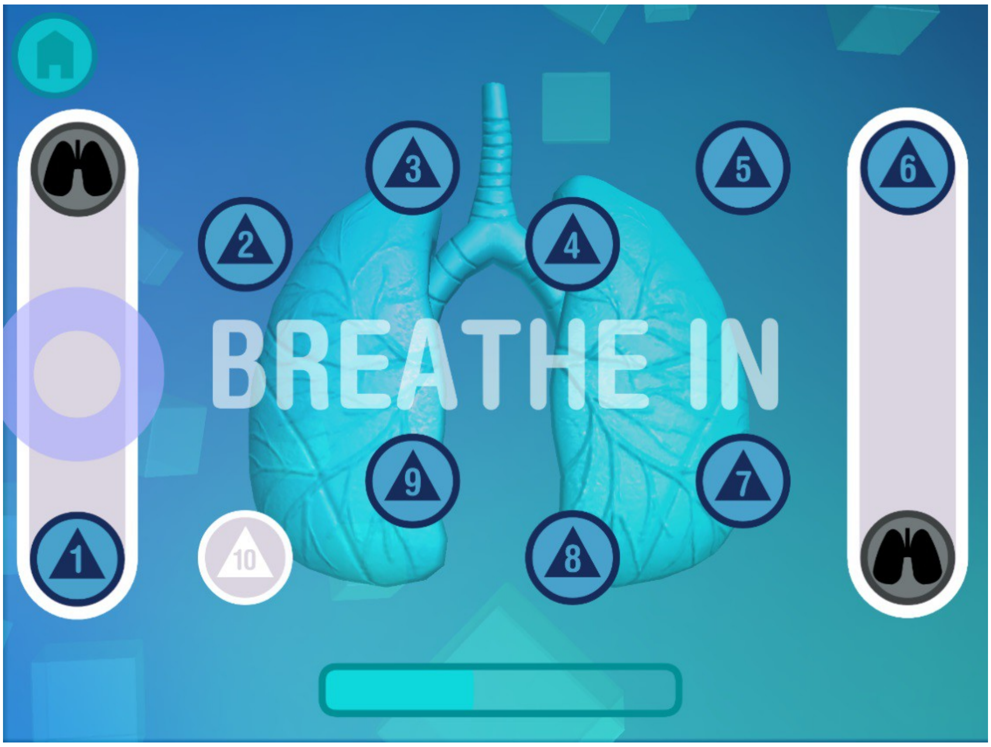

D.

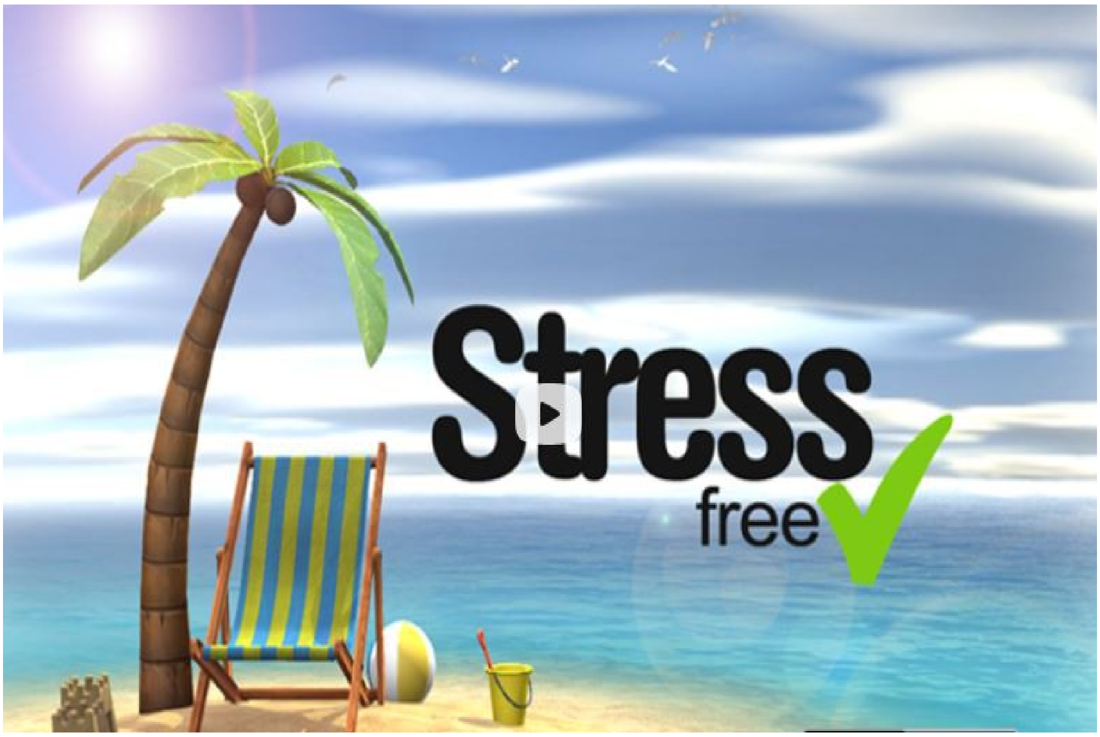

E.

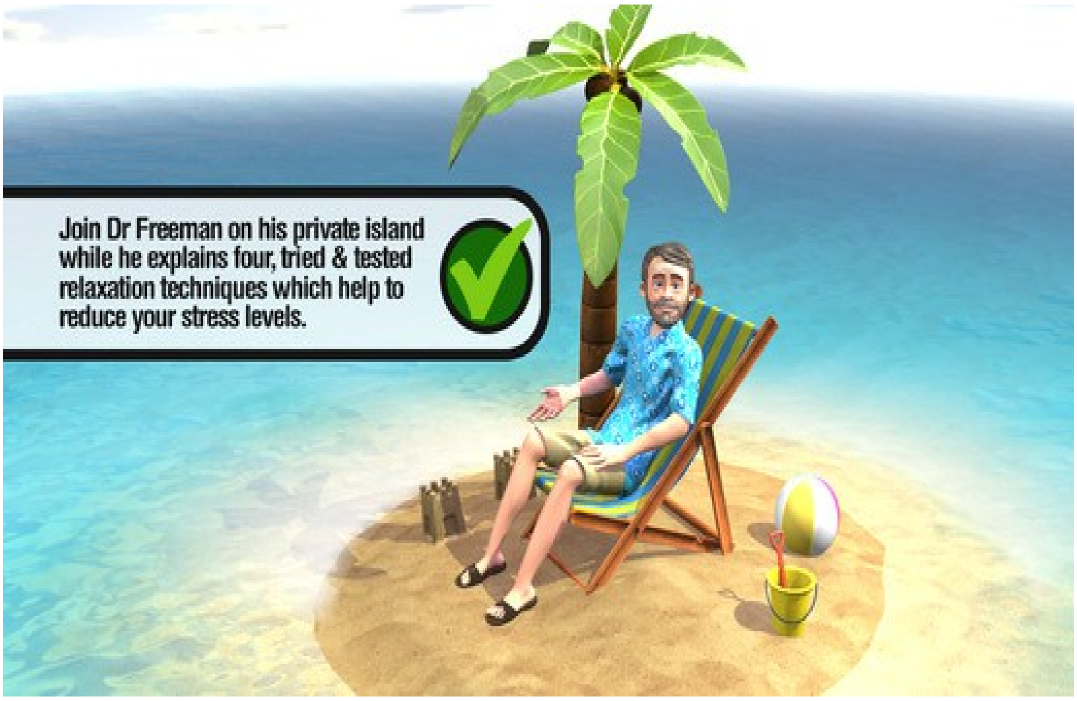
Join Dr Freeman on his private island while he explains four, tried & tested relaxation techniques which help to reduce your stress levels.

F.

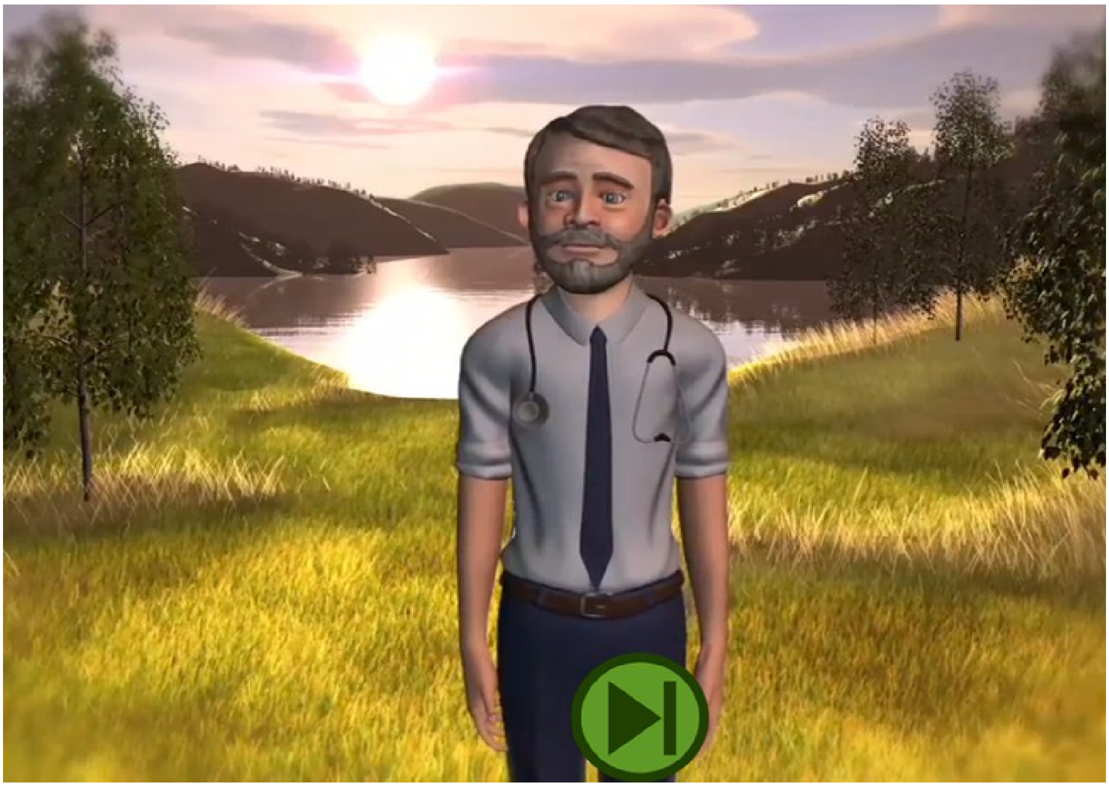

Supplement: Multimedia Appendix 2 [file jmir_v19i11e398_app2.pdf]
